# Assessing momentary relaxation using the Relaxation State Questionnaire (RSQ)

**DOI:** 10.1038/s41598-022-20524-w

**Published:** 2022-09-29

**Authors:** Sarah Steghaus, Christian H. Poth

**Affiliations:** 1 Biopsychology and Cognitive Neuroscience, Bielefeld University, Bielefeld, Germany; 2 Neuro-Cognitive Psychology and Center of Cognitive Interaction Technology (CITEC), Bielefeld University, Bielefeld, Germany

**Keywords:** Psychology, Health care

## Abstract

Stress is ubiquitous in everyday life and hazardous for mental and physical health. To prevent or ameliorate stress-related disease, relaxation exercises aim to counteract stress by inducing short-lasting states of relaxation on a regular basis. Critically, current assessments capture the mid- and long-term consequences of relaxation, however, cannot measure its short-term effects on an individual’s momentary psychological state. To address this problem, we developed the Relaxation State Questionnaire (RSQ). We assessed the psychometric quality of the questionnaire by investigating its item properties, reliability, and validity in an online study with 92 participants. Construct validity was examined through correlations with the Perceived Stress Questionnaire (PSQ; Fliege in 10.23668/PSYCHARCHIVES.2889, 2009). An exploratory factor analysis revealed four factors capturing the momentary state of muscle tension, sleepiness, cardiovascular activity, and general relaxation. In a second online study with 99 participants, we conducted a confirmatory factor analysis. Results revealed high item loadings (0.70–0.91), excellent reliability (α = 0.86) and excellent fit indices, and a good construct validity of the RSQ. These findings establish the RSQ as a tool to measure momentary states of relaxation. As such, the RSQ opens up research of the immediate subjective effects and the effectiveness of relaxation exercises.

## Introduction

Stress is a major risk factor for serious health problems, such as cardiovascular disease, diabetes, mental disorders such as anxiety and depression, and several other illnesses^1^. A vast number of different treatments have been developed to manage and reduce stress [e.g.,^2,3^, for an overview see^4^]. The most prominent treatments may broadly be classified as relaxation activities^5,6^. Assessing the effectiveness of stress management on an individual’s perceived psychological state requires adequate measures of subjective stress and relaxation. Such measures exist only for long-term effects of relaxation and perceived stress, but there are no adequate measures capturing the effects of relaxation over the short term. This is surprising, because the lack of adequate measures precludes research on the immediate effects and benefits of relaxation exercises. Here, we provide a new questionnaire, the Relaxation State Questionnaire (RSQ) to solve this problem. We show that the RSQ captures momentary states of relaxation efficiently, reliably, and validly. As such, the RSQ paves the way for research on the short-term effects and effectiveness of relaxation exercises and related interventions.

### Stress and long-term and short-term relaxation

Broadly defined, stress can be described as the body’s response to demand and pressure^7^. Those demands and pressures can take on various forms and are ubiquitous in everyday life: From financial stress, to stress with friends and family, to work stress and environmental stress [for an overview, see^7^]. As diverse as the sources of stress can be, are its consequences. For instance, stress hinders the immune system^8^, poses a risk for diabetes^9^, causes depression^10^, and is associated with a number of other diseases^1^. Throughout history, relaxation exercises have been developed as tools to relieve stress and prevent its negative ramifications^7^. Current exercises range from breathing exercises to progressive muscle relaxation (PMR), a relaxation exercise developed by Jacobsen^11^, that focuses on tensing and relaxing different muscle groups of the body^2^. To date, a number of studies delivered evidence that relaxation exercises such as PMR^12^, Tai-chi^13^, or guided imagery and hypnosis^14^ effectively reduce stress.

To benefit from relaxation exercises, one does not need years of experience and training. For example, 1 h of daily relaxation practice for 6 weeks can lead to a reduction of heart rate and blood pressure in students^15^. Furthermore, it was shown that relaxation moderated the relationship between occupational stress and mental health symptoms one month later^16^. In another study, the effects of only 3 consecutive days of mindfulness mediation were compared to sham meditation and a control group^17^. The authors found that the meditation exercise increased the mood and decreased both depression and heart rate. One study could even demonstrate positive effects of relaxation exercises (PMR and meditation) on anxiety measures after just one session^18^. Thus, taken together, it seems well-established that regular relaxation relieves stress and promotes health over the long-term, but there is also first evidence for beneficial effects of relaxation exercises over the short term.

### Measuring short-term relaxation

Assessing short-term effects and the short-term effectiveness of relaxation exercises requires to measure an individual’s current state of relaxation (or stress) just after the exercise. However, suitable questionnaires providing such an assessment of momentary states of relaxation from the individual’s point of view are still missing. This is because current measures focus on long-term consequences of relaxation that are more stable over time and thus affect an individual’s traits rather than situation-dependent states^19,20^. For instance, the Relaxation Inventory by Crist et al.^21^ or the Smith Relaxation States Inventory- Revised [SRSI-R;^22^] measure relaxation as a broad construct and more as a trait than as a state. For example, one item of the Relaxation Inventory is “I am worried about how much money I have.”. Items such as these refer to mental states over longer periods and thus cannot capture the momentary changes (e.g., induced by situational changes) in the state of relaxation. Also, the SRSI-R covers the dimension “Mystery”, referring to “feeling the deeper mystery of things beyond one’s understanding” [^23^, p. 1202], which also refers more to a personality trait and does not have a clear face validity regarding changes in relaxation states as they are necessary to evaluate relaxation techniques. Hites and Lundervold^24^ investigated the relationship between both questionnaires and a physiological observation of relaxation (including assessment of breathing and different body areas and muscles) and could not find the desired correlations. Thus, in sum, current tools are unable to assess the momentary states of relaxation, which means that questions of the immediate effects of relaxation exercises still remain unanswerable.

### The Relaxation State Questionnaire

To address these problems, we developed a new questionnaire, the Relaxation State Questionnaire (RSQ), which assesses a person’s momentary state of relaxation. Since it is specifically tailored to measure short-term relaxation states, the RSQ can be used to assess the immediate effectiveness of relaxation exercises (e.g., by comparing measures before and after a relaxation exercise). Here, we introduce the RSQ with its 10 items. In two studies, we investigated its psychometric properties, such as its item properties (mean values, item difficulties, and item discrimination), its factorial validity using both exploratory factor analysis (Study 1) and confirmatory factor analysis (Study 2) and its reliability. In addition, we validated the RSQ by obtaining correlations with specific subscales from an instrument capturing perceived stress (the German version of the Perceived Stress Questionnaire)^25^. That is, we assessed the scales “joy” and “tension”, whose items reflect some mental aspects of relaxation and the opposite state of relaxation, respectively. Thus, we predicted positive correlations of RSQ scales capturing relaxation with the joy scale, and negative correlations with the tension scale. An overview of our procedure in form of a research structure diagram can be found in Appendix C.

## Study 1: Exploratory factor analysis

### Method

#### Preregistration & power analysis

We preregistered our study to the Open Science Framework (OSF; https://osf.io/syext/?view_only=ce0d062f1cd24375ad4be13ab3a6c310). Furthermore, we conducted an a priori power analysis for the correlations and the exploratory factor analysis (EFA). A power analysis with the software G*Power [^26^, version 3.1] showed that for a power of .80, an alpha error probability of .05, and an effect size of .40 (intermediate effect) for the correlations, a sample size of 38 participants would be needed. For smaller correlations of around .30, 67 participants would be necessary, and for correlations of .20, 153 participants would be needed.

The number of participants for the exploratory factor analysis depends on the number of factors of the questionnaire, the factor loadings and the number of items loading on each factor^27^. Since all these parameters had yet to be determined for our questionnaire, the required minimum sample size could vary between 12 and 120 participants^27^.

Based on the power analyses and because our data collection had to take place within a time of 3 weeks, we planned and preregistered a sample size of minimally 40 and maximally 120 participants.

#### Sample

In total, 98 participants were recruited for the study. Psychology students at Bielefeld University were recruited through the Psychology Department’s participant pool and received course credit for participation. Furthermore, the study was distributed via social media groups to also obtain data from non-students.

Participants who did not report an age of at least 18 years, who were not at least “almost fluent” in German, and who indicated afterwards that they did not read and answer conscientiously, but merely clicked through, were excluded from the data set. Therefore, the final sample consisted of 92 participants (77 females, 14 males, and 1 person described themselves as “non-binary”).

The mean age was 28.4 years (*SD* = 13.9; *Mdn* = 23) with a range from 18 to 71 years. For their occupational status, 73 participants stated they were students, 10 employed, 2 job-seeking, and 6 participants stated they were retired.

All participants gave informed consent before the study. The study conformed to the ethical guidelines of the German Psychological Association (DGPs) and was approved by the ethics committee of Bielefeld University.

#### Measures

##### Relaxation State Questionnaire—RSQ

The goal was to develop a questionnaire that assesses the current relaxation state of a person. Since the questionnaire is supposed to be used in future studies and PMR experiments, the main goal was to assess the relaxational state of the body and mind with a focus on the muscles. Out of a review of different existing measurements^21,23,28^*,* and the theoretical considerations explained below, 10 items emerged that seem to fit this purpose. Table 1 displays the items and their English translation. Along with the 10 items, participants received the following instruction: “Below you will find a series of statements. Please read each statement and choose from the five answers the one that best describes, how much the statement is accurate to your feelings and condition right now, in this moment. There are no right or wrong answers. Please do not think too long about each statement and do not omit one.” (Instructions translated from German, the original instruction can be found in Appendix A) The five response categories were: not correct at all—rather not correct—neither nor—rather correct—entirely correct.

**Table 1:** Items and their English translation.

Item	Items in German	English Translation
1	Mein Atem ist schneller als gewöhnlich. (R)	My breathing is faster than usual. (R)
2	Mein Herz schlägt schneller als sonst. (R)	My heart is beating faster than usual. (R)
3	Meine Muskeln fühlen sich angespannt und verkrampft an (Hand zur Faust geballt; Kiefer angespannt; gerunzelte Stirn). (R)	My muscles feel tense and cramped (clenched fist and/or jaw; furrowed brow). (R)
4	Meine Muskeln fühlen sich entspannt an.	My muscles feel relaxed.
5	Meine Muskeln fühlen sich locker an.	My muscles feel loose.
6	Ich fühle mich sehr entspannt.	I'm feeling very relaxed.
7	Ich bin gerade vollkommen ruhig.	Right now, I am completely calm.
8	Ich fühle mich schläfrig und müde.	I'm feeling sleepy and tired.
9	Ich bin kurz davor einzunicken.	I'm about to doze off.
10	Ich fühle mich erfrischt und wach. (R)	I'm feeling refreshed and awake. (R)

Reverse-coded items are indicated by (R).

Effects of relaxation exercises are often classified into physiological and psychological aspects [see e.g.,^29,30^]. Physiological aspects of relaxation are rooted in the autonomic nervous system (i.e., sympathetic and parasympathetic nervous activity)^31^. Relaxation is thus connected with a lower sympathetic and a higher parasympathetic activation, resulting for example in a reduced cardiovascular activity, lower muscle tonus, and constricted pupils^32^. The first five items of the RSQ were thought to cover this physiological component of the relaxed state. For example, Question 4 states “My muscles feel relaxed.”. Three of the five questions specifically focus on the muscles, the other two on the breathing and the heart rate, which are two reliable indices for the level of stress hormones [^7^, Chapter 9] and the parasympathetic activation. Items 6–10 focus on the components of mental or psychological effects of relaxation. For example, Item 7 states “I am completely calm right now”. These items have a high face validity and are directed at participants subjective assessment of their own psychological state. We also included items regarding participants’ tiredness (Items 8–10). Tiredness or sleepiness could be seen as the extreme end of parasympathetic activation (and sympathetic deactivation). However, even though sleepiness is a dimension that can be found in other relaxation inventories as well^22^, sleepiness might be negatively correlated to relaxation^33^ and should thus be differentiated from relaxation. To judge their own sleepiness, participants do not have a physiological indicator as they have e.g., for their heart rate. For this reason, we consider it being part of the psychological/mental aspects.

##### Perceived Stress Questionnaire—PSQ

To validate the new questionnaire, the German version of the Perceived Stress Questionnaire (PSQ) by Fliege and colleagues^25^ was used. The questionnaire “assesses subjectively experienced stress independent of a specific and objective occasion” [^34^, p. 78] and had a Cronbach’s Alpha of 0.92 in a student population. Since the questionnaire targets “different facets of perceived stress” (p. 79) that are not all relevant in this context, only the subscales “joy” and “tension” were used. The scale tension “explores tense disquietude, exhaustion, and the lack of relaxation” (p. 83). Therefore, it should correlate negative with a higher score of relaxation of the RSQ. The joy subscale “is concerned with positive feelings of challenge, joy, energy, and security” (p. 83). Like the RSQ, each item is formulated as a statement (e.g., “I’m feeling tense.”) and participants are asked to rate on a four-point scale (almost never—sometimes—often—usually) how accurately the statement describes them, concerning the last two weeks of their lives. Note that this means that the PSQ and our RSQ assess their target constructs on somewhat different time scales. As mentioned above, the RSQ aims to assess momentary states of relaxation that varies over the short-term, whereas the PSQ assesses stress levels more in a “trait-like” fashion. Thus, the PSQ should therefore not be sensitive to short-term changes of relaxation. Nevertheless, it should still be able to provide a validation for the RSQ, because the individual long-term stress levels captured by the PSQ should impact on momentary stress or relaxation at a given point in time [e.g.,^20,35^].

#### Design and procedure

The online study took about 5–15 min to complete; however, participants had no time limit for the completion of the questions. After giving informed consent, the RSQ and the two subscales of the PSQ were presented to the participants in a random order with the above-mentioned instruction. The order of items within each questionnaire was also randomized. Every item had to be answered, otherwise participants were not able to continue with the questionnaire and complete the study. At the end, participants were asked for demographic information. The detailed procedure and phrasing of the study can be found in the study documentation on the OSF (https://osf.io/syext/?view_only=ce0d062f1cd24375ad4be13ab3a6c310).

#### Data analyses

Statistical analyses were performed in R [^36^, version 4.0.2]. A commented R-Script with all analyses can also be found on the OSF (https://osf.io/syext/?view_only=ce0d062f1cd24375ad4be13ab3a6c310)*.*

Inversely coded items were recoded with the recode function of the car package^37^. The Kaiser–Meyer–Olkin measure of sampling adequacy^38^ and the Bartlett's test of sphericity^39^ were computed to determine whether it was appropriated to perform a factor analysis on our data^40^. Item analysis and exploratory factor analysis were conducted with the describe function of the R-package psych^41^. For the item difficulty, the popularity index according to Dahl was calculated^42^. Item discrimination was determined with the alpha function of the psych package. Furthermore, to compute the reliability, the measurement model of the items must be determined first [see^43^]. Only then the reliability can be computed accurately. This was done with the lavaan package^44^. Reliability was then computed with the omega.tot function, using the Lambda4 and GPArotation packages^45,46^. Correlations between PSQ and RSQ were computed using the stats package^36^. Also, plots for the correlations were examined using the plot function to detect non-linear relations and statistical outliers.

## Results

### Exploratory factor analysis

Before computing the exploratory factor analysis, both, the Kaiser–Meyer–Olkin (KMO) measure, and the Bartlett test of sphericity were computed. The overall quality of the correlation matrix was .73 and thus above the cutoff of .5 proposed by Kaiser and Rice^47^. The Bartlett test of sphericity was significant (*p* < .001) and therefore justified a dimension reducing procedure.

An exploratory factor analysis of all 10 items was then performed in R. Since we expected factors to be correlated, an oblique rotation (promax) was conducted. The factors were interpreted based on the factor pattern matrix. This yielded a four-factor solution with three or two items loading on one factor, respectively. The factor loadings ranged between 0.44 and 1.07 and no cross loadings over 0.40 were found^48^ (see Table 2).

**Table 2:** Results from the factor analysis of the RSQ.

Item	Factor loading			
	1	2	3	4
**Factor 1: Muscle Score**				
5. My muscles feel loose.	**0.93**	0.13	− 0.10	0.08
4. My muscles feel relaxed.	**0.86**	0.00	− 0.08	0.07
3. My muscles feel tense and cramped (clenched fist and/or jaw; furrowed brow). (R)	**0.53**	− 0.06	0.05	0.09
**Factor 2: Sleepiness Score**				
8. I'm feeling sleepy and tired.	0.07	**1.07**	0.20	− 0.03
10. I'm feeling refreshed and awake. (R)	0.16	**0.75**	0.00	− 0.29
9. I'm about to doze off.	− 0.19	**0.44**	− 0.17	0.28
**Factor 3: Cardiovascular Score**				
1. My breathing is faster than usual. (R)	− 0.09	0.11	**0.89**	− 0.02
2. My heart is beating faster than usual. (R)	− 0.02	− 0.01	**0.71**	0.11
**Factor 4: General Relaxation Score**				
6. I'm feeling very relaxed.	0.15	− 0.01	− 0.06	**0.94**
7. Right now, I am completely calm.	0.14	− 0.04	0.21	**0.56**

The extraction method was principal axis factoring with an oblique rotation (promax with Kaiser normalization). Factor loadings above 0.40 are in bold. Reverse-scored items are denoted with (R).

Table 3 shows the correlations between the four found factors. Three factors (1, 3, and 4) were moderately positive correlated, Factor 2 (sleepiness score), however, correlated negatively with the others.

**Table 3:** Factor correlations for the four factors.

Factor	1	2	3	4
1. Muscle Score	–	− .42***	.38***	.43***
2. Sleepiness Score		–	− .49***	− .24*
3. Cardiovascular Score			–	.32**
4. General Relaxation Score				–

Factor correlations derived from EFA. All correlations reached significance.

*p < .05. **p < .01. ***p < .001.

### Item analyses

For the item analyses, means, standard deviations, and item difficulty were computed for all items. Furthermore, we computed the item’s discrimination power (using the correlation between the item score and the rest score of the scale) for those items who loaded on a factor with more than two items. All item properties can be found in Table 4.

**Table 4:** Item properties of the 10 items of the RSQ.

Item	*M*	*SD*	*P*	*rt*
My breathing is faster than usual. (R)	4.14	0.93	78.53	
My heart is beating faster than usual. (R)	4.02	0.96	75.54	
My muscles feel tense and cramped (clenched fist and/or jaw; furrowed brow). (R)	3.62	1.11	65.49	.56
My muscles feel relaxed.	3.33	1.06	58.15	.74
My muscles feel loose.	3.30	1.16	57.61	.73
I'm feeling very relaxed.	2.91	1.08	47.83	
Right now, I am completely calm.	3.32	1.09	57.88	
I'm feeling sleepy and tired.	3.13	1.18	53.26	.72
I'm about to doze off.	2.09	1.14	27.17	.44
I'm feeling refreshed and awake. (R)	3.33	1.03	58.15	.62

Rating scale for all items ranged from 1 to 5. *P* = Popularity index according to Dahl.

*rt* = Item discrimination (item rest correlation)*.* Reverse-scored items are denoted with (R).

### Measurement model and reliability

To adequately compute reliability, we tested the model fit of different measurement models by comparing the variance/covariance matrix of the four subscales of our data with the model implied matrices^43,49^.

We first tested the tau-congeneric model. The tau-congeneric model is the least restrictive measurement model, allowing different means, variances, and covariances within the matrix. This model simply assumes that the items of one subscale depend on the same latent variable, not necessarily to the same extent. The tau-congeneric model had an overall acceptable fit. Fit indices can be found in Table 5.

**Table 5:** Fit indices for both measurement models.

Fit Indices	Model	
	Tau-congeneric	Essential tau-equivalent
χ^2^/*df*	1.7	2
CFI	.95	.91
RMSEA	.09 [.04–.13]	.1 [.07–.14]
SRMR	.06	.1
AIC	2408.38	2417.07

CFI = comparative fit index; RMSEA = root-mean-square error of approximation (with 90% confidence interval in brackets); SRMR = standardized root-mean-square residuals; AIC = Akaike information criterion.

The next-restrictive measurement model to compare the tau-congeneric model to, is the essential tau-equivalent model. In the essential tau-equivalent model, the additional assumption that the covariances of the matrix are the same is implemented. This means, that the items of one subscale depend on the same latent variable to the same extend. The model fit became worse for this model in comparison to the tau-congeneric model (see Table 5). Hence, because of its better fit, we chose the tau-congeneric model for our data. Therefore, we computed Omega as a measurement for reliability, as proposed by Eid et al. [^43^, for a discussion of the different parameters see also^50^].

Reliability for the whole questionnaire was ω = 0.83. For the muscle score, reliability was ω = 0.83, for the sleepiness score ω = 0.79, for the cardiovascular score an ω = 0.74 was computed, and ω = 0.83 for the general relaxation score. Since the reliability can only be computed for subscale with more than two items, it could not be computed for the cardiovascular score and the general relaxation score. All computed reliability scores are above the cut-off values proposed for example by Reise and colleagues^51^ or Catalán^52^. Therefore, the reliability of the questionnaire can be rated as high.

### Correlations with PSQ

To examine the construct validity, first the total score for both the joy and the tension scale of the PSQ were computed according to the formula given by Fliege and colleagues^34^. Then, the scores were correlated with the four scales of the RSQ. No non-linear relations between the scores could be found when examining the plots. Table 6 shows the correlation between the RSQ and the PSQ scores.

**Table 6:** Correlations between RSQ and PSQ scales.

RSQ Scales	PSQ Scales	
	Tension	Joy
Muscle score	− .43***	.25**
Sleepiness score	.46***	− .45***
Cardiovascular score	− .20*	.24*
General relaxation score	− .33***	.22*

All correlations were tested one-tailed. *p < .05. **p < .01. ***p < .001.

All correlations remained significant after Bonferroni-Holm correction.

All correlations were significant and remained significant after adjusting for multiple testing with the Bonferroni-Holm correction [see^43^]. A table with *p* values and significance levels after the correction can be found in Appendix B. Table 6 shows the correlation between the scales of the PSQ and the RSQ. As hypothesized the tension scale and the RSQ scores (except for the sleepiness score) correlate negatively. For the joy scale, the assumed positive correlations were found, again except for the sleepiness score. However, since the sleepiness score correlated negative with the other scores of the RSQ in the EFA, we expected to find these reverse correlations (compared to the other RSQ scales) for the PSQ scales. Therefore, all correlations had the expected direction.

## Study 2: Confirmatory factor analysis

### Method

#### Sample

For the second experiment 106 students at Bielefeld University were recruited through the Psychology Department’s participant pool. Bachelor Psychology students received course credit for participation, however, the study was also open to non-psychology students. As in the first experiment, participants had to be at least 18 years old to participate. After excluding participants that did not give consent (i.e. aborted the study) or did not finish the study, 99 participants remained (85 females, 13 males, and one person who describes themselves as “diverse”). The mean age was 22.36 years (*SD* = 4.22, *Mdn* = 21) with a range of 18 to 49 years. All participants gave informed consent before the study. The study conformed to the ethical guidelines of the German Psychological Association (DGPs) and was approved beforehand by the ethics committee of Bielefeld University.

#### Design and procedure

The data acquisition was part of a larger study investigating effects of video streaming^53^ (for further details, see the preregistration of this study: https://osf.io/yx9zs/?view_only=bb80ae63bb904b739333194caa8021ab). The complete results of this study are going to be presented elsewhere. Participants in this study filled in the RSQ and two scales of the PSQ as a pre-test before the main part of the study began, and this data is used for our confirmatory factor analysis of the RSQ.

#### Data analyses

As in the first study, statistical analyses were performed in R (version 4.1.1). The commented R-Script with all analyses can be found on the OSF (https://osf.io/syext/?view_only=ce0d062f1cd24375ad4be13ab3a6c310)*.*

For the confirmatory factor analysis and the computation of the measurement model the lavaan package was used^44^.

## Results

Before computing the factor analysis, the Kaiser–Meyer–Olkin (KMO) measure and the Bartlett test of sphericity were computed. The overall quality of the correlation matrix was .81 and thus above the cutoff of .5 proposed by Kaiser and Rice^47^, indicating that the data is suited for factor analyses. The Bartlett test of sphericity was significant (*p* < .001) and therefore indicates that the data is factorable. Also, there was no missing data, meaning for all participants, all items were answered. The test for multivariate normality revealed that the data was not normally distributed (for both kurtosis and skewness *p* < .001), therefore the maximum likelihood estimation with robust standard errors was used for the CFA^54^.

**Figure 1:**
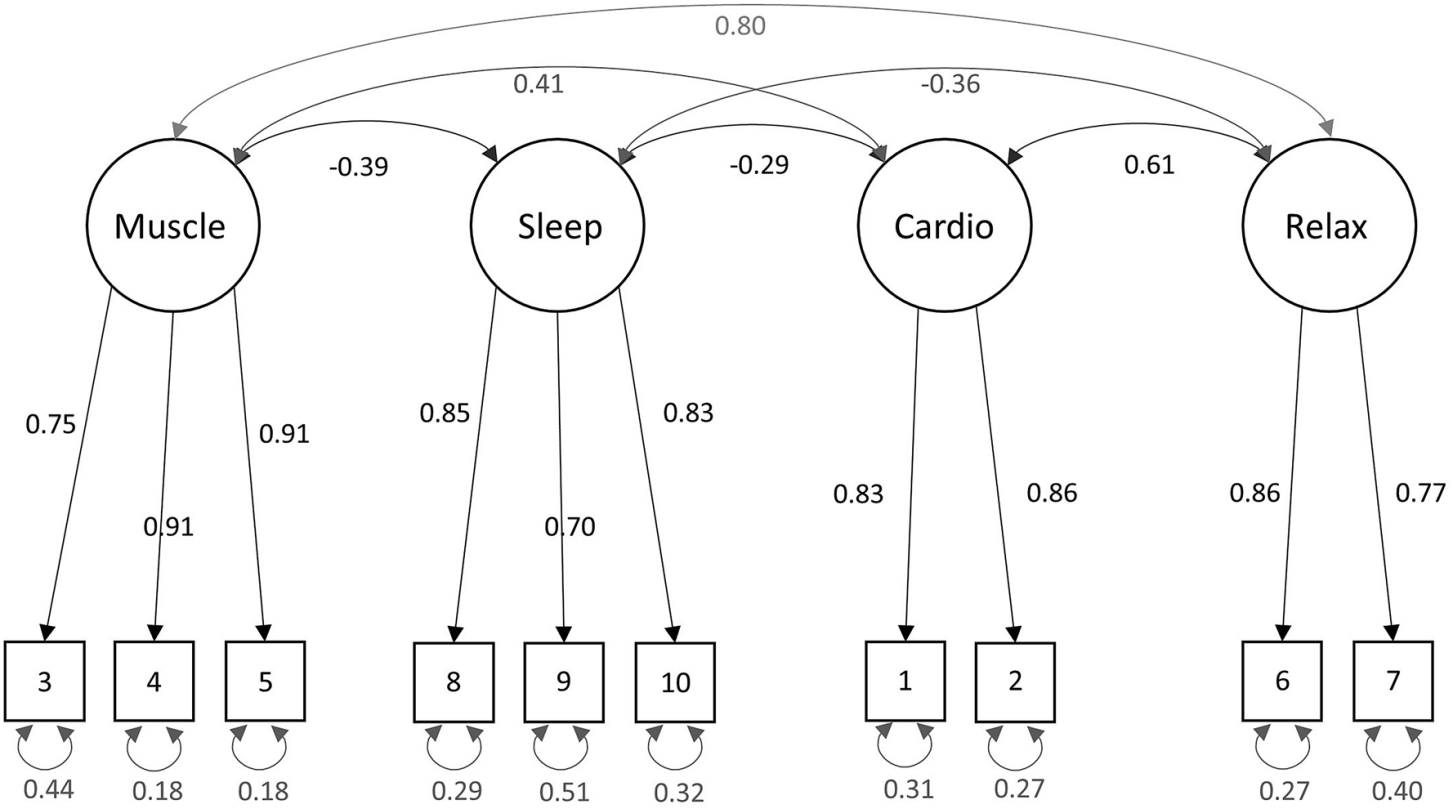
Structure model with parameter estimates. Note. Items are numbered as indicated in Table 2. All parameter estimates are standardized.

The confirmatory factor analysis with the 10 items was performed with lavaan, using the proposed model from the exploratory factor analysis. The CFA revealed high factor loadings between 0.7 and 0.91 for the items (see Table 7). Figure 1 shows the structure of the RSQ with loadings and correlations between the four factors. Fit indices for the CFA, with χ^2^/*df* = 0.98 and a comparative fit index (CFI) = 1, indicate an excellent fit [^54^, p. 74 f.]. Following the guidelines from Jackson et al.^55^, further fit indices were computed and can be found in Table 8.

**Table 7:** Results from the confirmatory factor analysis.

Item	Factor loading			
	1	2	3	4
**Factor 1: Muscle Score**				
1. My muscles feel tense and cramped (clenched fist and/or jaw; furrowed brow). (R)	0.75			
2. My muscles feel relaxed.	0.91			
3. My muscles feel loose.	0.91			
**Factor 2: Sleepiness Score**				
1. I'm feeling sleepy and tired.		0.85		
2. I'm about to doze off.		0.70		
3. I'm feeling refreshed and awake. (R)		0.83		
**Factor 3: Cardiovascular Score**				
1. My breathing is faster than usual. (R)			0.83	
2. My heart is beating faster than usual. (R)			0.86	
**Factor 4: General Relaxation Score**				
1. I'm feeling very relaxed.				0.86
1. Right now, I am completely calm.				0.77

All loadings are standardized loadings. Reverse-scored items are denoted with (R).

**Table 8:** Fit indices for different measurement models.

Model	Fit indices				
	χ^2^/*df*	CFI	RMSEA	SRMR	AIC
Tau-congeneric (CFA)	0.98	1	0 [0–.08]	.04	2354.47
Ess. tau-equivalent	0.95	1	0 [0–.07]	.05	2347.5
Tau-equivalent	4.22	.78	.17 [.14–.19]	.19	2468.99
Ess. tau-parallel	1.36	.97	.6 [0–.1]	.06	2361.7

CFA = confirmatory factor analysis; Ess. = essential; CFI = comparative fit index; RMSEA = root-mean-square error of approximation (with 90% confidence interval in brackets); SRMR = standardized root-mean-square residuals; AIC = Akaike information criterion.

The tau-congeneric model was already evaluated with the model underlying the CFA.

### Measurement model and reliability

To adequately compute reliability, we also tested the model fit of different measurement models. Fit Indices for four different models can be found in Table 8. Comparison of the fit indices revealed that the best fitting model is the essential tau-equivalent model. Therefore, Cronbach’s Alpha can be used to determine reliability^56,57^.

The overall reliability of the RSQ (α = 0.86) can be classified as good^57^. Computation of reliability for each scale individually revealed equally satisfying scores of α = 0.89 (muscle scale), α = 0.83 (sleepiness scale), α = 0.83 (cardiovascular scale), and α = 0.80 (general relaxation scale).

## Discussion

The aim of this study was to develop and validate a new questionnaire that assesses the momentary state of relaxation, the Relaxation State Questionnaire (RSQ). The factorial validity showed a four-factor solution for our 10 items. Item properties and reliability were also computed. To further validate the questionnaire, we correlated it with the two subscales “joy” and “tension” of the German version of the Perceived Stress Questionnaire. We further tested the four-factor model with a confirmatory factor analysis, which yielded high factor loadings, an excellent model fit, and a high reliability. The results of our analyses showed that the RSQ is a reliable and valid instrument with good item properties. As such, the RSQ paves the way for research on momentary states of subjective relaxation and thus of the immediate effects and effectiveness of relaxation exercises.

### Item properties and factory validity

The item properties of the questionnaire were all satisfactory. All items were used to their full range, except for item 2 (“My heart is beating faster than usual.”), which only had a range from 2 to 5. Furthermore, no item displayed ceiling or floor effects. The item difficulty ranged from 47.83 to 78.53, with the exception of item 9 (“I’m about to doze off.”), whose difficulty was the lowest with 27.17. Therefore, all items have medium difficulties and are suited for the questionnaire [see e.g.,^42^].

The exploratory factor analysis proposed a four-factor solution for the items. With only 10 items in total, this means that there are two factors with only two items each. Even though this means that item discrimination could not be computed for these scales, all items had very high loadings on their respective factors (0.44 to 1.07) and no cross loadings above 0.4 were detected. This indicates that the items fit very well with their respective factors and other factor solutions would not seem appropriate. The confirmatory factor analysis validated this structure and yielded even higher factor loadings, ranging from 0.70 to 0.91. Even though, the four-factor solution was not expected beforehand, the different scores of the RSQ may be very useful to differentiate relaxation and effects of different relaxation exercises: The muscle score can be useful for PMR exercises, the cardiovascular score for breathing exercises, and the sleepiness score can function as a manipulation check to test whether exercises actually relaxed participants and patient or just made them drowsy or fatigued.

Reliability was computed for the overall questionnaire and each scale specifically. Omega reached 0.83 for the questionnaire in the EFA data set, exceeding the requirements proposed by Catalán^52^ and thus revealing a reliable measurement. Confirming this with the CFA data set, a high reliability of α = 0.86 was computed. Item discrimination values ranged between .56 and .74, indicating that all items can adequately differentiate between participants with high and low levels of relaxation [cf.^42^]. Therefore, the item selection for the questionnaire succeeded and no items should be excluded.

### Construct validity

To further determine construct validity for our new questionnaire, correlations with the Perceived Stress Questionnaire^25^ were computed. Since the sleepiness score (Factor 2) is negatively correlated with the other factors, the four scales/factors were correlated independently with the two subscales of the PSQ. As hypothesized, there was a negative correlation between the first factor (containing the items for the muscles) and the scale “tension”. This validates the intended objective for these items.

Also, the items correlated negative with the subscale “joy”. As one would expect from the factor correlations, the items on the sleepiness scale correlate negative with “tension” scale and negative with the “joy” scale. Construct validity of our questionnaire can therefore be assumed.

### Assessing momentary states of subjective relaxation

To successfully measure momentary states and changes in the subjective feeling of relaxation, self-report measures, such as this questionnaire, are essential. They capture the phenomenal state of relaxation that is private and only (if at all) accessible by report. Also, self-reports are known to have a high accuracy, predictability, and utility^58^. Indirect indicators of relaxation (e.g., physiological measures) cannot adequately represent the participants’ state of mind and inner subjective emotional world. After all, the most direct way to assess relaxation is to ask if one feels relaxed. As such, even though one might argue that two items per scale in our questionnaire are too few, these items have the highest face validity (e.g., Item 6 “I’m feeling very relaxed.”) and should thus be kept in the questionnaire. This is consistent with their high loadings on their respective factors.

Since completing a questionnaire is a cognitive activity which might interfere with the current state of relaxation, we aimed for a short and efficient, and yet reliable questionnaire. While the 10 items do cover several aspects of relaxation and are suited for various relaxation exercises, the overall time needed to complete the questionnaire is very short. In highly dynamic situations, for example, within cognitive experiments in which questionnaires would interrupt the task too much, the usage of only one or two score of the RSQ can even be considered. This procedure would allow a most efficient measurement of certain aspects of the relaxation state without inferring with the relaxation effect itself.

### Assessing the effectiveness of relaxation exercises

Currently, there are no reliable and valid tools for assessing momentary states of relaxation from the participant’s point of view. As a consequence, short-term changes in these states cannot be assessed. This is a problem for the empirical evaluation of all relaxation exercises because their short-term effects and their effectiveness cannot be quantified. The RSQ fills this gap and provides a reliable and valid assessment of relaxation, which allows the evaluation of relaxation exercises such as progressive muscle relaxation^2,59^, mindfulness meditation^3,60^, and other meditation techniques.

### Limitations and future research

One might ask if the sample sizes of this study was sufficient for our exploratory factor analysis. This seems to be the case, since it has been shown that sample sizes as ours can yield reliable results^27,61,62^. This is also supported by the high factor loadings for the questionnaire, which indicate that only a smaller number of participants was needed for reliable results. The confirmatory factor analysis replicated our initial findings, again demonstrating the good reliability and confirming the factor structure and validity of the RSQ. Thus, taken together, this indicates that our present results are robust, and they establish the RSQ as a reliable and valid tool for assessing states of relaxation.

It is an open question how sensitive the RSQ is for manipulations of the relaxation state by interventions which target subjective relaxation. This question provides an interesting avenue for future studies that manipulate the state of relaxation experimentally and monitor the resulting change in relaxation. In this way, the RSQ could be further validated using experimental manipulations known to tap into specific and theory-based components of relaxation. Moreover, even though traditional assessments of relaxation focus more on its long-term aspects^21,22^, it would be interesting to investigate whether these assessments converge or diverge with the state measures provided by the RSQ. The subscales of the RSQ could also provide a useful tool to closely compare the effects of competing relaxation exercises^29^. Moreover, the RSQ opens up to study how *self-reported* relaxation (measured by the RSQ) and *physiological measures* of relaxation such as heart rate or pupillometry relate to one another. Recent studies could show that there seems to be a correlation between self-report and arousal^63^, however, these effects are possible mediated by a number of factors^64^, calling for more specific follow-up studies.

## Conclusions

We developed a questionnaire assessing the momentary state of subjective relaxation (Relaxation State Questionnaire, RSQ). Item properties, factor loadings, and reliability were all satisfactory in two independent samples. In addition, the construct validity could be established by means of correlation with an established instrument. With 10 items, the questionnaire is brief and can be easily implemented in a range of research settings. Taken together, the RSQ paves the way for research on short-lasting states of relaxation and opens up investigations of the immediate effectiveness of relaxation exercises.

## Supplementary Information

Supplementary Information.

## Data Availability

The datasets generated and analyzed during the current study are available on the Open Science Framework (OSF) [https://osf.io/syext/?view_only=ce0d062f1cd24375ad4be13ab3a6c310].
