# Supplementary material for: Assessing momentary relaxation using the Relaxation State Questionnaire (RSQ)
Source: Sci Rep. 2022 Sep 29;12:16341. doi: 10.1038/s41598-022-20524-w (PMC9522935; doi:10.1038/s41598-022-20524-w)
Supplement: Supplementary file 1 — Supplementary Information. [file 41598_2022_20524_MOESM1_ESM.pdf]

Appendix A

Original Instructions of the RSQ (in German)

Im Folgenden finden Sie eine Reihe von Feststellungen. Bitte lesen Sie jede durch und wählen Sie aus den fünf Antworten diejenige aus, die angibt, wie sehr die Feststellung auf Ihr Befinden jetzt, in diesem Moment, zutrifft. Es gibt keine richtigen oder falschen Antworten. Überlegen Sie bitte nicht lange und lassen Sie keine Frage aus.

Appendix B

Table With Bonferroni-Holm Corrected *p* Values

Table B1

Correlations Between PSQ and RSQ with Bonferroni-Holm Correction

Correlation	*p* value	Significance level after correction
Tension & cardiovascular score	.03044	.05
Joy & general relaxation score	.01676	.025
Joy & cardiovascular score	.01194	.01667
Joy & muscle score	.008106	.0125
Tension & general relaxation score	.0006075	.01
Tension & muscle score	.0000107	.008333
Joy & sleepiness score	.000004298	.00714
Tension & sleepiness score	.000002403	.00625

Note. Correlations were ordered by *p* value, then for every correlation, the new significance level was computed by the formula given by Eid et al. [43] with ($\alpha = .05$). All correlations remained significant.

Appendix C

Research Structure Diagram

Figure C1

Research Structure Diagram of Procedure

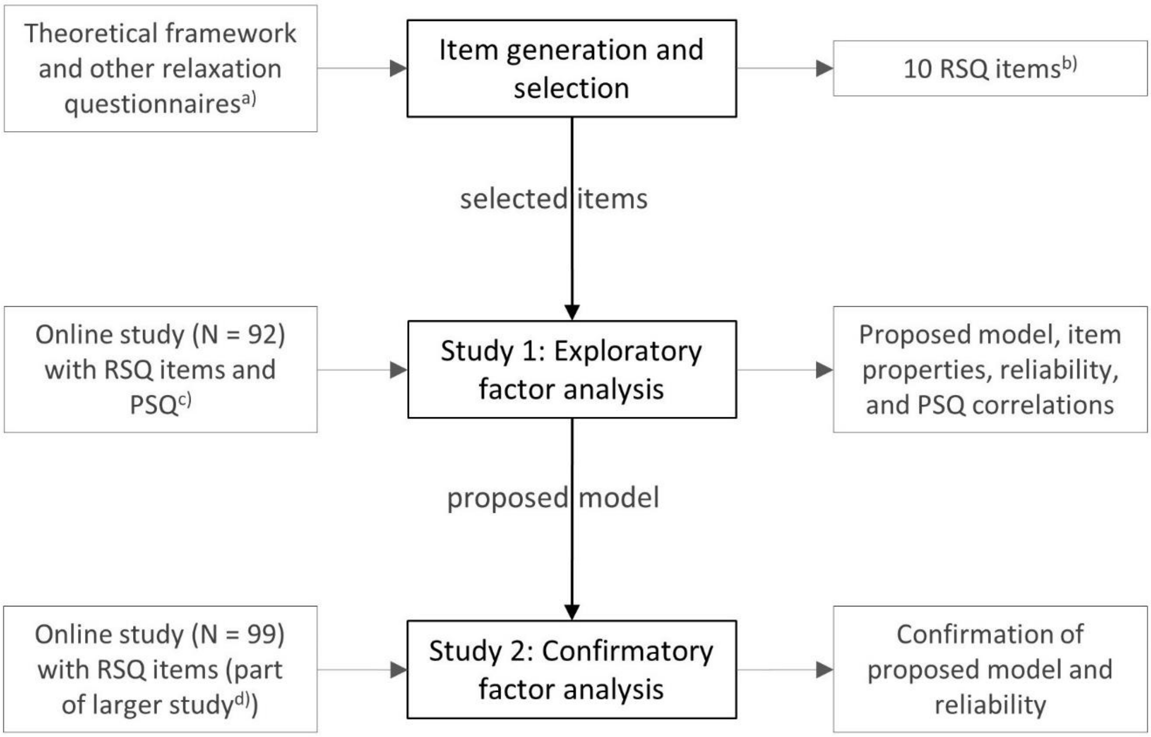

Note. Diagram depicting steps in the development of the Relaxation State Questionnaire (RSQ) with inputs and outcomes for each step.

[a)] [21,22,28]. [b)] For list of items see Table 1. [c)] PSQ = Perceived Stress Questionnaire [25]. [d)] [53].
